# Diffusion- and Susceptibility Weighted Imaging Mismatch Correlates With Collateral Circulation and Prognosis After Middle Cerebral Artery M1-Segment Occlusion

**DOI:** 10.3389/fneur.2021.660529

**Published:** 2021-07-22

**Authors:** Zhihua Xu, Zhenhua Tong, Yang Duan, Dengxiang Xing, Hongyan Song, Yusong Pei, Benqiang Yang

**Affiliations:** ^1^Department of Radiology, TongDe Hospital of Zhejiang Province, Hangzhou, China; ^2^Department of Radiology, Center for Neuroimaging, General Hospital of Northern Theater Command, Shenyang, China; ^3^Department of Scientific Research, General Hospital of Northern Theater Command, Shenyang, China; ^4^General Hospital of Northern Theater Command Training Base for Graduate, Jinzhou Medical University, Shenyang, China; ^5^Center for Medical Data, General Hospital of Northern Theater Command, Shenyang, China; ^6^Department of Radiology, General Hospital of Northern Theater Command, Shenyang, China

**Keywords:** diffusion weighted imaging, susceptibility weighted imaging, collaterals, prognosis, hypointensity vessel sign prominence

## Abstract

**Objective:** To explore the relation between diffusion-weighted and susceptibility weighted imaging (DWI-SWI) mismatch and collateral circulation or prognosis in patients with occluded M1 segments of middle cerebral artery (MCA).

**Methods:** We enrolled 59 patients with MCA M1-segment occlusion for a retrospective review of baseline clinical and imaging data. As markers of circulatory collaterals, prominent laterality of posterior (PLPCA) and anterior (PLACA) cerebral arteries on magnetic resonance angiography (MRA) studies and a hyperintense vessel sign (HVS) on T2 fluid-attenuated inversion recovery (FLAIR) images were collectively scored. The extent of acute cerebral infarction was then quantified on DWI, using the Alberta Stroke Program Early CT Score (DWI-ASPECTS). Hypointensity vessel sign prominence (PVS) was also evaluated by SWI and similarly scored (SWI-ASPECT) to calculate DWI-SWI mismatch [(DWI-ASPECTS) – (SWI-ASPECTS)], ranging from −10 to 10 points.

**Results:** DWI-SWI mismatch showed significant associations with PLPCA, PLACA, HVS prominence, and collective collateral scores (all, *p* < 0.05). National Institutes of Health Stroke Scale (NIHSS), DWI-SWI mismatch, and DWI-ASPECTS also differed significantly according to patient prognosis (good vs. poor) after MCA M1-segment occlusion (*p* < 0.05). In binary logistic regression analyses, NIHSS and DWI-SWI mismatch emerged as independent prognostic factors (*p* < 0.05).

**Conclusions:** Collateral circulation may be an important aspect of DWI-SWI mismatch, which in this study correlated with prognostic outcomes of MCA M1-segment occlusion.

## Introduction

Although occlusion of the middle cerebral artery (MCA) is a major cause of anterior circulation strokes, clinical outcomes may vary as a function of collateral circulation. The anastomosing networks of ipsilateral anterior or posterior cerebral artery and leptomeningeal artery serve to compensate for perfusion deficits. Magnetic resonance imaging (MRI) is a common means of assessing such collaterals *in vivo*. In earlier studies, prominent laterality of posterior (PLPCA) and anterior (PLACA) cerebral arteries observed by magnetic resonance angiography (MRA) and a hyperintense vessel sign (HVS) on T2 fluid-attenuated inversion recovery (FLAIR) images have shown clear associations with both collateral vessels and patient prognosis (1, 2).

Susceptibility weighted imaging (SWI) is a unique technology widely applied in recent years for *in vivo* venous assessments. As a consequence, we now better appreciate the venous changes occurring after strokes and their impact on clinical outcomes (3–5). Some studies have linked diffusion- and susceptibility weighted imaging (DWI-SWI) mismatch to the penumbra after MCA occlusion (6, 7). However, the implications of collateral circulation for DWI-SWI mismatch have not been fully explored as yet. The present investigation was undertaken to examine the relation between DWI-SWI mismatch and collateral circulation or prognosis in patients with occluded M1 segments of MCA.

## Materials and Methods

### Patient Selection

The study protocol received approval of our Institutional Review Board, each enrollee or his/her legally authorized representative providing written informed consent at the onset. All clinical and imaging data collected came from patients presenting between January 2016 and August 2019. Inclusion criteria were as follows: (a) occluded M1 segment of MCA, confirmed by MRA or computed tomography (CT) angiography; (b) acquisition of T1- (T1WI) and T2- (T2WI) weighted imaging, T2-FLAIR imaging, diffusion-weighted imaging (DWI), SWI, and MRA within 24 h after admission; and (c) first-time stroke. Exclusion criteria were the following: (a) other brain abnormalities, such as tumor, infection, trauma, or vascular malformation; (b) poor image quality; and (c) incomplete clinical data.

### Clinical Data Collection

Baseline parameters of study subjects, including age, sex, and risk factors for stroke (i.e., hypertension, diabetes mellitus, atrial fibrillation, active smoking, hyperlipidemia, and hyperhomocysteinemia), were culled from medical records. We also recorded National Institutes of Health Stroke Scale (NIHSS) rankings upon admission, and patients were scored by modified Rankin Scale (mRS) at 90 days after discharge (good prognosis, mRS <3). All patients did not treat with revascularization, but got aggressive supportive treatment.

### Magnetic Resonance Imaging Protocol

All patients underwent multimodal MRI studies on a 3.0 T scanner (Discovery MR750; GE Healthcare, Chicago, IL, USA) using an eight-channel phased-array head coil. The settings used were as follows: (a) DWI [repetition time (TR), 3,000 ms; echo time (TE), 65.3 ms; b-value, 1,000 s/mm^2^]; (b) SWI [TR, 27 ms; TE, 20 ms; flip angle, 10°; slice thickness, 2 mm; intersection gap, 0 mm; field of view (FOV), 24 × 24 cm^2^; matrix number, 512 × 512]; (c) T2-FLAIR (TR, 8,800 ms; TE, 94 ms; inversion time, 2,500 ms; slice thickness, 5 mm; intersection gap, 1 mm; FOV, 24 × 24 cm^2^; matrix number, 512 × 512); and (d) 3D-TOF MRA (TR, min; TE, 2.3 ms; flip angle, 20°; slice thickness, 0.6 mm; intersection gap, 0 mm; FOV, 24 × 24 cm^2^; matrix number, 256 × 256). Moreover, the time from stroke onset to imaging was calculated.

### DWI-SWI Mismatch Determinations

Two neuroradiologists separately reviewed all images (ZHX and HYS reviewed the data of DWI-SWI mismatch, YD and YSP reviewed the data of collaterals), resolving any disagreements through consensus (BQY and two reviewers). The neuroradiologists receive anonymized imaging data and did not know which MR modality corresponds to the same patients' other MR modalities and clinical data. An interval time of a week was set when evaluated two different MR modality. First, we determined whether acute cerebral infarction was attributable to unilateral M1-segment MCA occlusion. We then assessed the extent of infarction on DWI images, using the standard Alberta Stroke Program Early CT Score (DWI-ASPECTS) to quantify changes (range, 0–10). A score of 0 indicated diffuse cerebral infarction in the region of MCA blood supply, no new cerebral infarction scored as 10.

**Figure 1:**
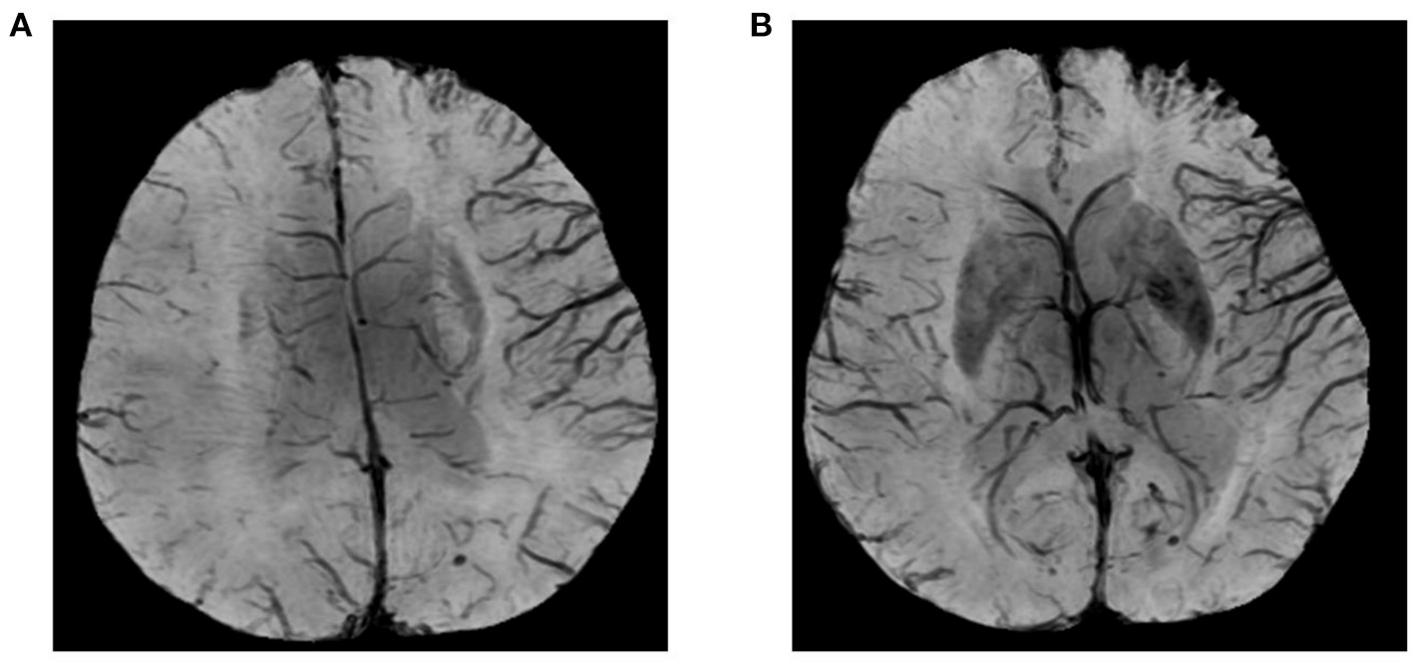
Images illustrating prominent hypointensity vessel sign (PVS) and SWI-ASPECTS: blood supply in area of right middle cerebral artery (M1, M2, M3, insula, M4, M5, and M6) showing prominent PVS; and thalamostriate venous none-dilatation (SWI-ASPECT of 3).

SWI data were processed by minimum-intensity projection (slice thickness, 10 mm) to detect venous changes, namely prominent hypointensity vessel sign (PVS). PVS was defined as veins greater in number or diameter within affected (vs. contralateral) hemispheres (Figure 1). To quantify PVS, draining veins of anterior circulation were divided into 10 regions and scored as above (SWI-ASPECTS). Each PVS-positive region called for one point subtracted from the 10-point maximum. Because veins of lenticular nucleus, caudate nucleus, and posterior limb of internal capsule are drained by the thalamostriate vein, we subtracted three points if thalamostriate vein was dilated and PVS positive. A normal SWI earned a score of 10, whereas 0 indicated diffuse PVS positivity.

To determine DWI-SWI mismatch (range, −10 to 10), we subtracted respective scores: (DWI-ASPECTS) – (SWI-ASPECTS). A score of −10 indicated massive cerebral infarction in the region supplied by MCA, with obstructed venous drainage due to parenchymal edema or decompensating cerebral ischemia despite an increasing oxygen extraction fraction (OEF). A score of 10 corresponded with no new cerebral infarction or active compensation for cerebral ischemia through OEF increase.

### Gauging Collateral Circulation

**Figure 2:**
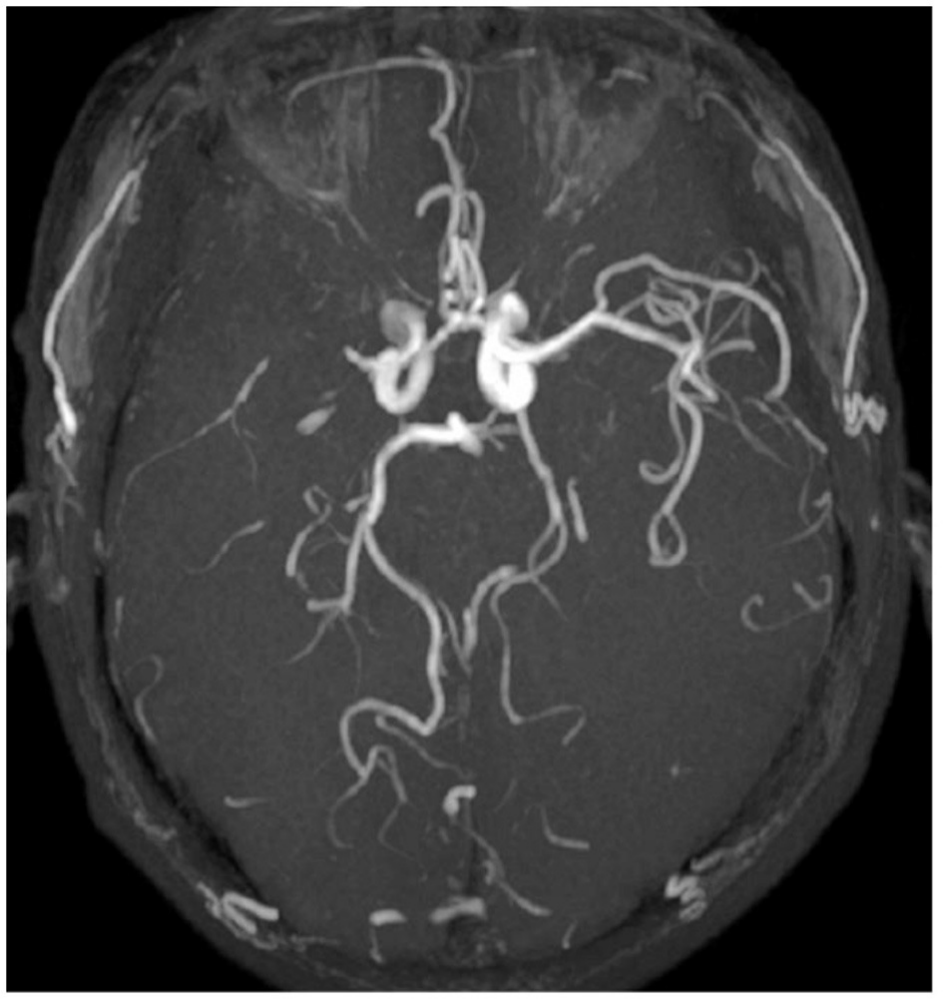
Images illustrating prominent laterality posterior (PLPCA) and anterior (PLACA) cerebral arteries: M1 segment of right middle cerebral artery occluded, right posterior cerebral artery dilated, and P4 segment visible, with visible P3 segment of left posterior cerebral artery (PLPCA); and greater visibility of right anterior cerebral artery vs. distal end of left anterior cerebral artery (PLACA).

**Figure 3:**
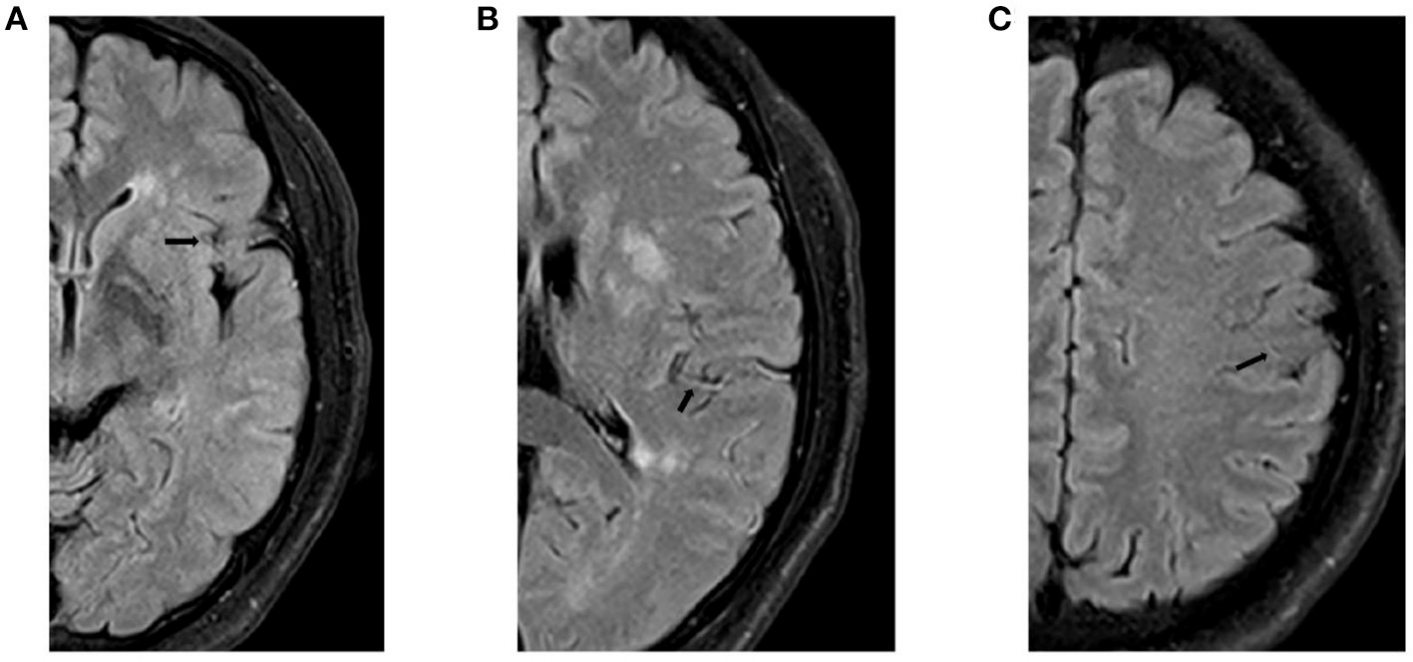
Hyperintense vessel sign (HVS): **(A)** Score of 1, restricted to Sylvian fissure; **(B)** Score of 2, limited to Sylvian fissure and cerebral sulci of temporo-occipital junction; and **(C)** Score of 3, extension to cerebral sulci of frontoparietal lobe, in addition to locations scored as 1 or 2.

PLPCA and PLACA were identifiable on MRA images reconstructed via maximum-intensity projection. Each was distinguishable as greater cerebral arterial segmentation within affected (vs. contralateral) hemispheres (Figure 2). HVS was detectable on T2-FLAIR images, reflecting the status of leptomeningeal collateral vessels. A scoring system (range, 0–3) was used to quantify HVS as follows: 0, absence of HVS; 1, HVS limited to Sylvian fissure; 2, HVS limited to Sylvian fissure and cerebral sulci of temporo-occipital junction; and 3, extension to cerebral sulci of frontoparietal lobe, in addition to locations scored as 1 or 2 (Figure 3). A score of 0 or 1 signified poor collaterals, with good collaterals scored as 2 or 3. As a collective score (range, 0–3), PLPCA, PLACA, and HVS prominence each contributed one point.

### Statistical Analysis

We expressed categorical variables as frequencies and percentages, continuous normally distributed data as means and standard deviations (SDs), and non-parametric data as medians and interquartile ranges (IQRs). The Kolmogorov-Smirnov test was used to perform normality test. To assess differences, we subjected normally distributed data to Student's *t*-test; categorical variables to Chi-square test; and non-parametric data to Mann-Whitney *U*-test or Kruskal-Wallis test. Pearson or Spearman correlation analysis was invoked to explore the relation between DWI-SWI mismatch and collateral vessels. Factors independently linked to prognosis were discerned by logistic regression. All computations were driven by standard software (SPSS for Windows v20; IBM Corp, Armonk, NY, USA), setting significance at *p* < 0.05.

## Results

In this study, there were 59 enrollees (men, 38) with MCA M1-segment occlusions (mean age, 65 ± 10 years), 31 presenting with PLPCA, 16 with PLACA, and 30 with HVS prominence. The median of collective collateral scoring was 1 (IQR, 0–2); and at 90 days after discharge, prognosis was good (mRS <3) in 33 patients.

### DWI-SWI Mismatch and Collateral Circulation

**Figure 4:**
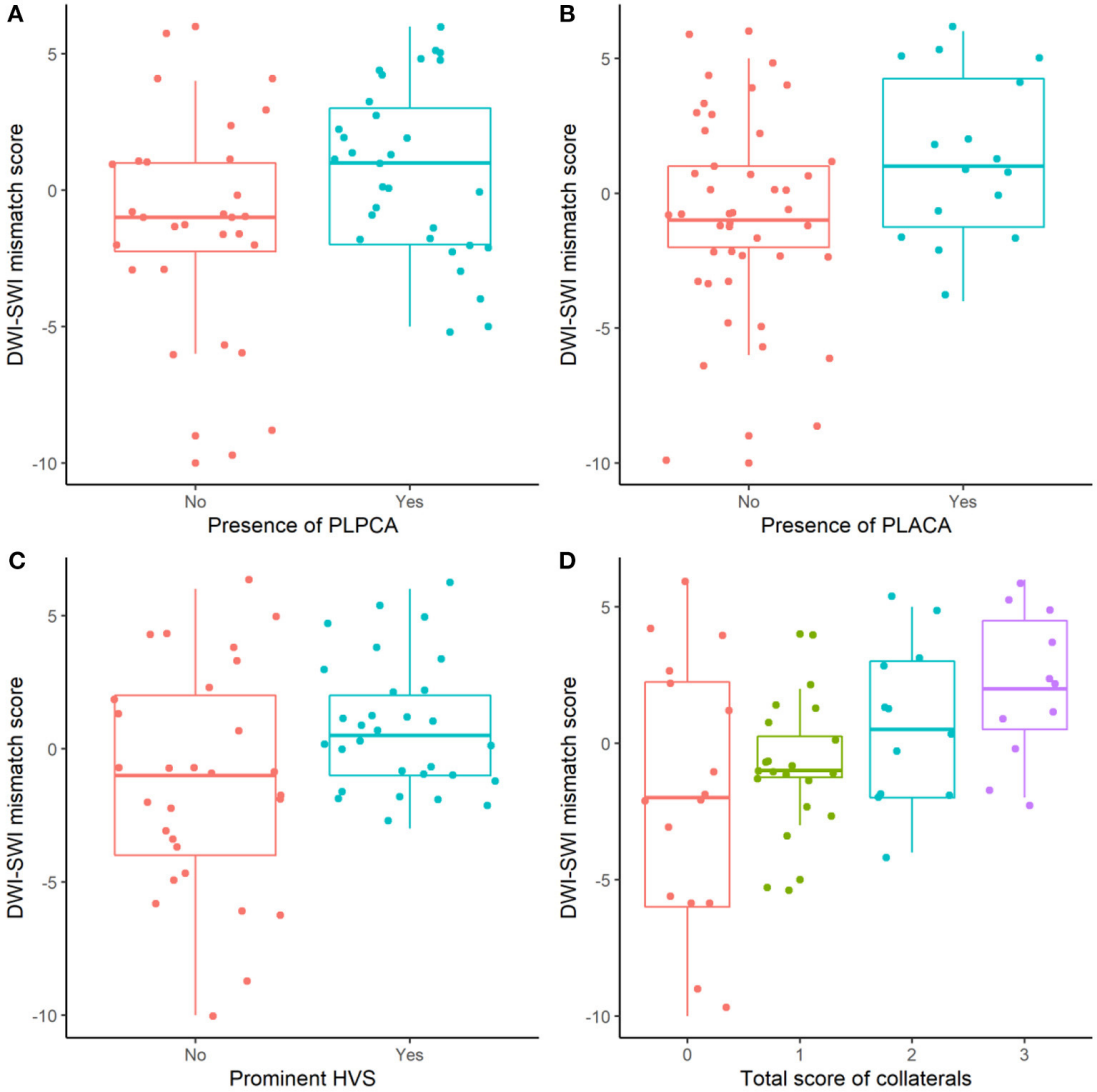
DWI-SWI mismatch and collateral circulation: significantly differing DWI-SWI mismatch in absence vs. presence of PLPCA, PLACA, or HVS prominence, and in collective collateral scoring (all, *p* < 0.05).

**Figure 5:**
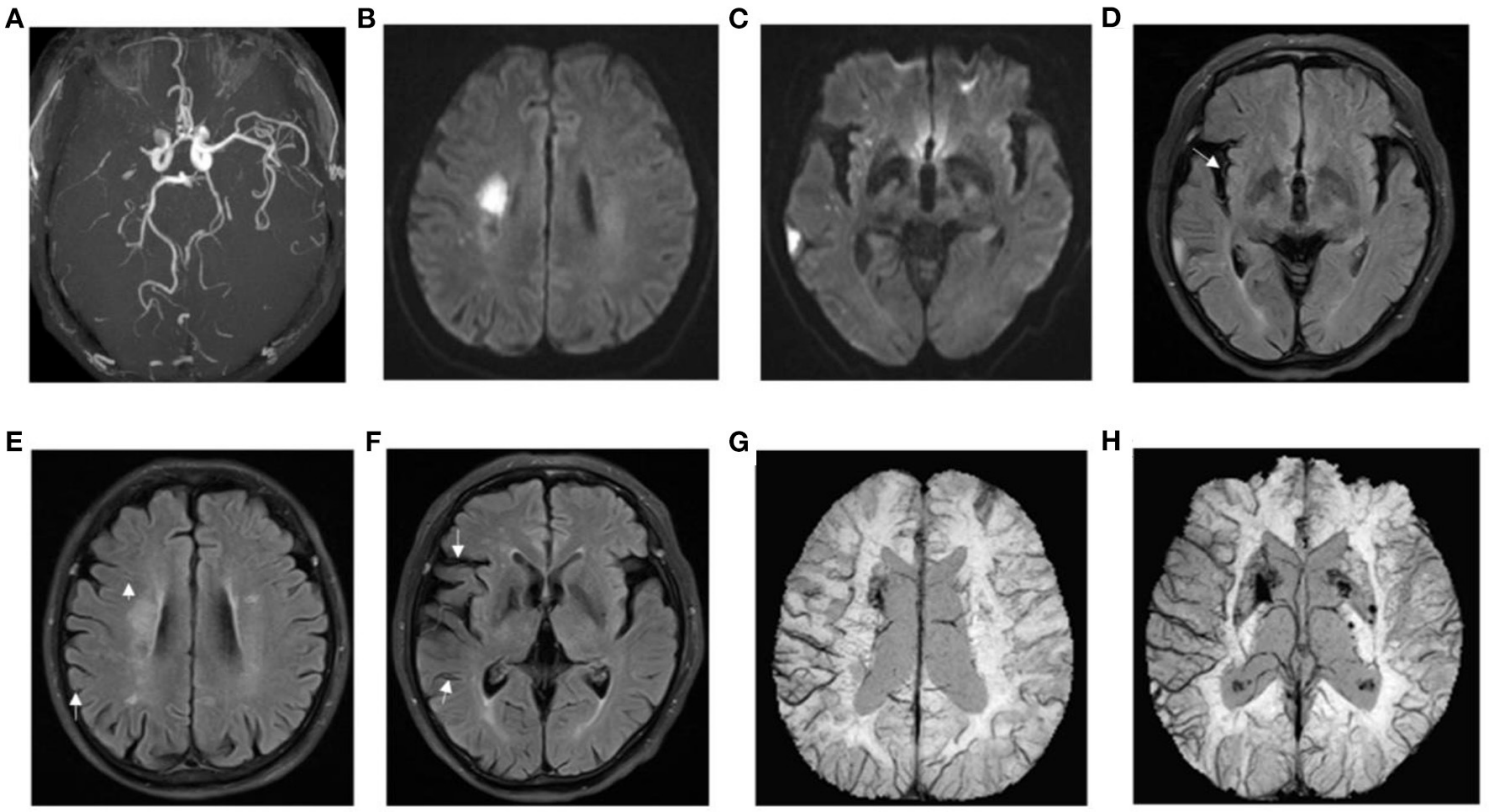
Presentation of a typical patient. A 62-year-old man with left limb weakness for 5 h. **(A)** right prominent laterality posterior (PLPCA) and anterior (PLACA) cerebral arteries; **(B,C)** diffusion-weighted imaging indicated acute cerebral infarction, and the DWI-ASPECTS was 7; **(D–F)** T2 fluid-attenuated inversion recovery (FLAIR) images showed hyperintense vessel sign (HVS), and a HVS score of 3; **(G,H)** susceptibility weighted imaging demonstrated prominent hypointensity vessel sign (PVS), and SWI-ASPECTS was 3. DWI-SWI mismatch score was 4. The patient had a favorable prognosis with a mRS of 1 at 90 days.

The relation between DWI-SWI mismatch and collateral circulation is shown in Figure 4. Mean DWI-SWI mismatch differed significantly in patients with (1 ± 3) and without (−1 ± 4) PLPCA [*p* = 0.037; Pearson coefficient (*r*) = 0.273]; in patients with (1 ± 3) and without (−1 ± 3) PLACA (*p* = 0.035; *r* = 0.275); and in patients with (1 ± 3) and without (−1 ± 4) HVS prominence (*p* = 0.021; *r* = 0.299). Moreover, DWI-SWI mismatch score correlated significantly with collective collateral scoring (*p* = 0.003; *r* = 0.384). Images of a typical patient was shown in Figure 5.

### DWI-SWI Mismatch and Patient Prognosis

In Table 1, baseline clinical and imaging data of the study population are presented by prognostic status after MCA M1-segment occlusion. Age, sex, stroke risk factors (hypertension, diabetes mellitus, atrial fibrillation, active smoking, and atrial fibrillation), PLACA, PLPCA, HVS prominence, collective collateral scoring, and SWI-ASPECTS did not differ significantly in prognostic subsets (good vs. bad; all, *p* > 0.05); although there were significant differences in NIHSS, DWI-SWI mismatch, and DWI-ASPECTS (all, *p* < 0.05). In binary logistic regression analyses (Table 2), NIHSS and DWI-SWI mismatch emerged as independent factors associated with patient prognosis (both, *p* < 0.05).

**Table 1:** Baseline clinical and imaging data of patients with MCA M1-segment occlusion, shown by prognostic status.

	**Poor prognosis**	**Good prognosis**	***P*-value**
	***n* = 25**	***n* = 34**	
Age, years	67 ± 9	63 ± 9	0.107
Male sex	16 (64.0)	22 (64.7)	1.000
**Stroke risk factors**			
Hypertension	16 (64.0)	23 (67.6)	0.989
Diabetes mellitus	10 (40.0)	8 (23.5)	0.284
Atrial fibrillation	5 (20.0)	3 (8.8)	0.393
Active smoking	13 (52.0)	16 (47.1)	0.911
NIHSS	12 ± 5	5 ± 5	<0.001
PLACA, yes	7 (28.0)	9 (26.5)	1.000
PLPCA, yes	12 (48.0)	19 (55.9)	0.737
Prominent HVS, yes	11 (44.0)	19 (55.9)	0.523
Collective collateral scoring			0.788
0	7 (28.0)	9 (26.5)	
1	10 (40.0)	10 (29.4)	
2	4 (16.0)	8 (23.5)	
3	4 (16.0)	7 (20.6)	
DWI-SWI mismatch	−2 ± 3	1 ± 3	<0.001
DWI-ASPECTS	4 ± 2	6 ± 2	<0.001
SWI-ASPECTS	6 ± 2	5 ± 3	0.055
Time^*^, hours	11.5 ± 3.3	11.9 ± 2.1	0.569

*Data expressed n (%) or as mean ± SD*.

*MCA, middle cerebral artery; NIHSS, National Institutes of Health Stroke Scale; PLACA, prominent laterality of anterior cerebral artery; PLPCA, prominent laterality of posterior cerebral artery; HVS, hyperintense vessel sign; DWI, diffusion-weighted imaging; SWI, susceptibility weighted imaging; ASPECTS, Alberta Stroke Program Early CT Score*.

Time*: *the time from stroke onset to imaging*.

**Table 2:** Prognostic indices in patients with MCA M1-segment occlusion.

	**Odds ratio**	**95% Confidence interval**		***P*-value**
		**Low**	**High**	
DWI-ASPECTS	1.098	0.576	2.092	0.776
NIHSS score	0.818	0.691	0.967	0.019
DWI-SWI mismatch	1.630	1.089	2.438	0.017

*MCA, middle cerebral artery; DWI, diffusion-weighted imaging; NIHSS, National Institutes of Health Stroke Scale; ASPECTS, Alberta Stroke Program Early CT Score; SWI, susceptibility weighted imaging*.

## Discussion

In this investigation, DWI-SWI mismatch showed significant association with collateral circulation and prognosis in patients presenting with occluded M1 segments of MCA. Hence, higher degrees of DWI-SWI mismatch indicate greater potential for good collateral flow (i.e., presence of PLPCA, PLACA, and HVS) and satisfactory outcomes.

Advances in SWI technology, particularly the introduction of intravenous deoxygenated hemoglobin as an endogenous contrast agent, have enabled clear delineation of even small intracranial veins. In recent years, the intracranial venous changes that follow ischemic insults have attracted much clinical attention, generating more clinical data and guidelines for clinical treatment of ischemic cerebrovascular diseases (8–10). PVS is a common marker of venous changes in the aftermath of stroke (11). Researchers currently have tendered two explanations for PVS. One is that the ischemic state is inclined to produce vasodilation, thus increasing vascular volume (4). The other is that blood oxygen supply and demand are unbalanced in areas of low brain tissue perfusion, causing a rise in deoxygenated hemoglobin, a fall in oxygenated hemoglobin, and a decline in intravascular signals (12).

Studies conducted more recently indicate a significant correlation between PVS and abnormal intracranial perfusion in this setting. Indeed, a number of sources have demonstrated good correlation between SWI-ASPECT and mean transit time (MTT)-ASPECT (13, 14). Given the evidence on DWI-MTT perfusion mismatch, it is not unreasonable to view DWI-SWI mismatch as a biomarker for predicting hyperacute cerebral ischemic penumbra and guiding stroke recanalization treatment clinically. According to one study of DWI-SWI mismatch and ischemic penumbra in cerebral infarction, DWI-SWI mismatch signals areas of “misery perfusion,” marked by low perfusion and high OEF. These are sites where damage to brain tissue is reversible and may be resolved through appropriate clinical intervention. The establishment of collateral circulation is a critical phenomenon that improves the prognosis. However, the precise relation between DWI-SWI mismatch and collateral circulation remains unclear.

Once the M1 segment of MCA is occluded, the circle of Wills offers no compensatory option. Above this point, collateral circulation is conferred primarily by ipsilateral anterior or posterior cerebral arteries and the leptomeningeal artery. HVS on T2-FLAIR image indicates slow anterograde or retrograde leptomeningeal collaterals (15, 16). PLPCA (PLACA) on MRA indicates the presence of collateral flow via the leptomeningeal anastomoses from the ipsilateral PCA(ACA) to the MCA in patients with proximal occlusion of the MCA (1). Herein, we have shown that in conjunction with M1-segment MCA occlusion, PLPCA, PLACA, and HVS are all correlates of DWI-SWI mismatch. Thus, collateral circulation may be considered an important aspect of DWI-SWI mismatch. Upon occlusion of the M1 segment, cerebral blood flow abruptly declines in the region of brain supplied by MCA. The posterior and anterior cerebral arteries of ipsilateral brain then enlarge, accentuating blood flow and harnessing leptomeningeal anastomoses to provide a rich compensatory collateral circulation. At the same time, the OEF in cerebral blood vessels increases as a compensatory measure, causing accumulation of deoxyhemoglobin (manifested as PVS). Through these complementary mechanisms, the blood oxygen level of brain tissue is restored and brain damage prevented or contained. Consequently, there is mismatch between DWI-ASPECTS (high) and SWI-ASPECTS (low).

Although we did confirm a significant association between DWI-SWI mismatch and patient outcomes, our data do not support a relation between collateral circulation and good prognosis (mRS <3) at 90 days. This particular finding is nevertheless aligned with a report by De Havenon et al., claiming that good collaterals may correlate with reduced ischemic core growth but are not a factor in neurologic outcomes (17). On the other hand, DWI-SWI mismatch does appear related to both collaterals and prognosis, jointly representing DWI-ASPECTS and SWI-ASPECTS. It not only embodies the status of collaterals but also reflects responses to or compensatory effects of ischemia, underscoring effective (rather than failed) compensation. Consequently, DWI-SWI mismatch may be a better measure of collateral circulation in this setting. We did encounter a few patients with good collaterals and low NIHSS scores on admission who experienced poor clinical outcomes, ostensibly tied to DWI-SWI mismatch in areas of “misery perfusion.” As time goes by, the microcirculation may evolve, leading to collapse of established collaterals (18). Without timely intervention, collaterals alone do not constitute a firewall against continuance of acute-phase cerebral infarction. Intervention must be expeditious to restore compromised brain tissue and improve the prognosis.

Moreover, with respect of ischemic stroke due to large artery occlusion, DWI-SWI mismatch also attracted some other researchers' attention. These studies were focusing on the relationship of DWI-SWI mismatch to DWI-perfusion mismatch and clinical prognosis (7, 19), while this study is highlighting the correlation between DWI-SWI mismatch and collaterals. Moreover, we use routine MRI sequences, which are available to most hospital MRI labs affiliated to stroke units. Thus, it could be directly replicable by clinicians.

The present study has certain limitations, chiefly the small sampling of patients and the single-center environment. Also, because severely affected patients often are ill-suited for MRI studies or often produce poor images, there was an element of selection bias. Larger numbers of patients from multiple centers should be evaluated going forward. We did not perform digital subtraction angiography, given the high costs entailed and visibility issues prohibiting direct detection of collateral status.

## Conclusion

Collateral circulation may be an important aspect of DWI-SWI mismatch, which in this study showed a significant association with patient prognosis after MCA M1-segment occlusion.

## Data Availability Statement

The original contributions presented in the study are included in the article/supplementary material, further inquiries can be directed to the corresponding author/s.

## Ethics Statement

The studies involving human participants were reviewed and approved by General Hospital of Northern Theater Command. The patients/participants provided their written informed consent to participate in this study.

## Author Contributions

ZX, ZT, and YD conceived the project idea. BY provided critical suggestions for the experiments design. ZX, ZT, DX, YP, and HS collected the imaging and clinical data. ZX, HS, YD, YP, and BY provided the imaging analysis. ZX, ZT, YD, and BY wrote the paper. YD and BY supervised the project. All authors contributed to the article and approved the submitted version.

## Conflict of Interest

The authors declare that the research was conducted in the absence of any commercial or financial relationships that could be construed as a potential conflict of interest.
